# Genome sequence and analysis of *Escherichia coli* production strain LS5218

**DOI:** 10.1016/j.meteno.2017.10.001

**Published:** 2017-11-02

**Authors:** Jacqueline M. Rand, Gina C. Gordon, Christopher R. Mehrer, Brian F. Pfleger

**Affiliations:** a Department of Chemical and Biological Engineering, University of Wisconsin, Madison, United States; b Microbiology Doctoral Training Program, University of Wisconsin, Madison, United States

**Keywords:** *E. coli* K-12, Genome sequence, Polyhydroxyalkanoate, Metabolic engineering

## Abstract

*Escherichia coli* strain LS5218 is a useful host for the production of fatty acid derived products, but the genetics underlying this utility have not been fully investigated. Here, we report the genome sequence of LS5218 and a list of large mutations and single nucleotide permutations (SNPs) relative to *E. coli* K-12 strain MG1655. We discuss how genetic differences may affect the physiological differences between LS5218 and MG1655. We find that LS5218 is more closely related to *E. coli* strain NCM3722 and suspect that small genetic differences between K-12 derived strains may have a significant impact on metabolic engineering efforts.

## Highlights

• *Escherichia coli* LS5218 genome sequence is presented.
• *E. coli LS5218* is more closely related to *E. coli* NCM3722 than MG1655.
• E. coli LS5218 has 17 large and 74 small differences from MG1655.

## 1 Introduction

*Escherichia coli* strain LS5218 is frequently studied for the production of polyhydroxyalkanoates (PHAs) from mixtures of sugars and organics acids (Agnew et al., 2012, Nduko et al., 2012, Salamanca-cardona et al., 2014). LS5218 is selected because of two commonly cited differences from other *E. coli* strains – mutations in *fadR* (*fadR601)* and *atoC* (*atoC(c)*). The *fadR601* disrupts expression of FadR thereby deregulating the *fad* genes that encode enzymes responsible for β-oxidation (Fujita et al., 2007). AtoC is an activator of the *atoDAEB* operon, encoding enzymes required for catabolism of acetoacetate and other short-chain organic acids (Lioliou et al., 2005, Theodorou et al., 2011). The *atoC(c)* mutation alters the regulator and causes constitutive expression and upregulation of the *atoDAEB* operon (Jenkins and Nunn, 1987, Matta et al., 2007). The mutations in *E. coli* LS5218 allow for increased uptake and utilization of a wider array of fatty acid chain-lengths and make it well-adapted for the engineering of short chain length-co-medium chain length (SCL-co-MCL) copolymers and poly(3-hydroxybutyrate-co-3-hydroxyvalerate) [P(3HB-co-3HV)] (Rhie and Dennis, 1995, Tappel et al., 2012). Despite its widespread use in PHA production studies, the genome sequence of *E. coli* LS5218 has not been made publicly available. This is in part due to the common assumption that it is a close relative of the sequenced *E. coli* K-12 strain MG1655.

**Fig. 1:**
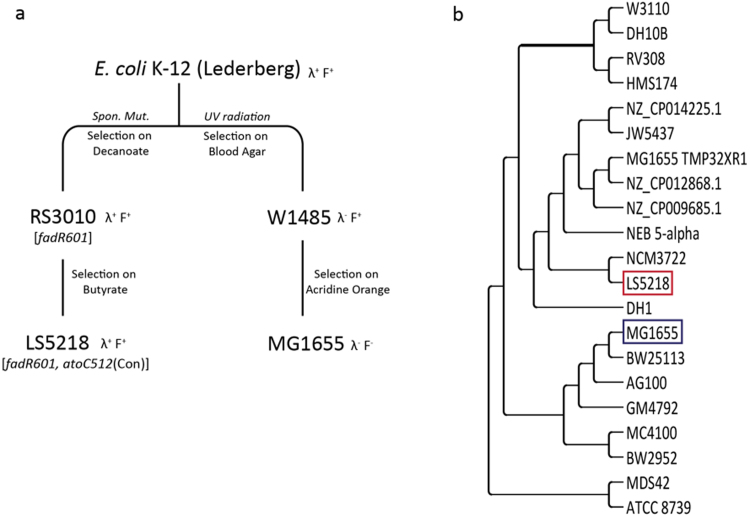
Comparison of LS5218 and MG1655. A. Diagram of the published derivation paths for LS5218 and MG1655. B. Pan-genome phylogenetic tree for *E. coli* K-12 strains. Strains listed with published name, or accession number if a published name was not listed. Spon. Mut., spontaneous mutation.

While a variety of *E. coli* strains are widely used by researchers, the history of their isolation is not as widely known. The original *E. coli* K-12 was isolated in 1922 and deposited in the Stanford University strain collection in 1925 (Neidhardt et al., 1996). The two main wild-type K-12 strains, WG1 from J. Lederberg and EMG2 from Clowes and Hayes, are subcultures of the Stanford K-12 strain. The published derivation of *E. coli* LS5218 involved a two-step screening of spontaneous mutants on selective media (Fig. 1**A**) (Spratt et al., 1981). Strain RS3010 was a spontaneous mutant of the Lederberg *E. coli* K-12 strain selected for growth on decanoate, to isolate a mutant with upregulated β-oxidation gene expression (Simons et al., 1980). Strain LS5218 was generated as a spontaneous mutant of RS3010 selected for on butyrate in order to isolate a strain with the ability to metabolize SCL fatty acids (Spratt et al., 1981). *E. coli* MG1655 was derived from an original K-12 isolate from the Lederberg lab through a two-step process designed to cure out the bacteriophage lambda (UV radiation and blood agar selection) and the F plasmid (acridine orange) (Blattner et al., 1997a). *E. coli* MG1655 and *E. coli* LS5218 appear to be derived from the same *E. coli* K-12 isolate (the Lederberg K-12 strain), but differences in their derivation histories convinced us to sequence *E. coli* LS5218 to know the exact genetic background of this production strain. Here, we report the genome sequence of *E. coli* LS5218 and an analysis of its content relative to *E. coli* MG1655 and a closer relative *E. coli* NCM3722.

## 2 Results and discussion

*E. coli* LS5218 genomic DNA was sequenced using paired end reads on a HiSeq. 2500 System, then assembled into 121 contigs using SPAdes (Bankevich et al., 2012). The draft genome was deposited in GenBank (GCA_002007165.1) and the NCBI Prokaryotic Genome Annotation Pipeline (PGAP) automatically assigned annotations. Using the annotated protein features for LS5218, we generated a phylogenetic tree comparing LS5218 with 21 completely sequenced *E. coli* K-12 derivatives using the Bacterial Pan Genome Analysis pipeline (BPGA) (Chaudhari et al., 2016). The pan genome analysis compiled a set of core genes common in all strains, accessory genes present in at least two or more strains, and unique genes only found in a single strain. The algorithm used this information to perform phylogenetic clustering of the *E. coli* K-12 derivative strains based on their variable gene content (Fig. 1**B**). The phylogenetic clustering shows that LS5218 is more closely related to the newly sequenced strain *E. coli* NCM3722 than it is to MG1655.

Next, we compared the newly assembled LS5218 genome with the *E. coli* MG1655 reference genome to evaluate the genetic relationship between the two strains. We used the Mauve genome alignment software (Darling et al., 2004, Darling et al., 2010) to align the genome contigs for LS5218 against MG1655 and found 17 large differences in the form of gene insertions, deletions and genome rearrangements between the two strains (Table 1). We also performed single nucleotide polymorphism (SNP) and indel analysis with FreeBayes (Garrison and Marth, 2012), and found 74 small differences listed in Table 2. Through this analysis, we confirmed the presence of mutations in *fadR* and *atoC*.

**Table 1:** Table of Large insertions and deletions between MG1655 and LS5218.

**Location**	**MG1655**	**LS5218**	**Comments**
**257905‐258680**	IS1I	No insert	Intact *crl* gene in LS5218
**279599‐291070**^b^	No insert	Deletion – recombination at insA elements	Deletion of 11 genes of cryptic prophage CP4–6
**574587‐575785**^b^	insH1	No insert	Intact *nmpC* gene in LS5218
**687850‐689049**^b^	insH1	No insert	IS5 upstream of *gltIJKL* operon in MG1655
**807329**^b^	No insert	λ phage	Wild type λ phage in LS5218
**916878**^a^	No insert	Insertion in *ybjD*	Premature stop codon
**1299498–1300697**	IS5U	No insert	upstream of *oppA*
**1878573**^b^	No insert	IS5	Disrupted *yeaP* gene in LS5218
**1978505–1979294**^b^	IS1	Tn1000	Insertions upstream of *flhDC*
**2101742–2102945**^b^	IS5	No insert	Intact *wbbL* gene in LS5218
**2110297–2128593**^b^	No insert	IS1 and 18 kb deletion	Deleted: *rfbA, rfbD, rfbB, galF, wcaM, wcaL, wcaK, wzxC, wcaJ, cpsG, cpsB, wcaI, fcl, gmd, wcaF*
**2170165–2171620**^b^	IS3	No insert	Intact *gatR* in LS5218
**3130145**^a^	IS5	3.5 kb insert	Inserted: fatty acyl-AMP ligase, short chain dehydrogenase, ACP binding site family protein
**3365549–3366752**^b^	IS5	No insert	Intact *yhcE* gene in LS5218
**4480807**^b^	No insert	IS1	Disrupted *yjgN* gene in LS5218
**4498173–4499513**^b^	IS2	No insert	Insertion in MG1655 between pseudogenes in KpLE2
**F Plasmid**^b^	No	Yes	

a Similar position but different from reported mutation in NCM3722 (Lyons et al., 2011).

b , Mutation also reported for NCM3722 (Lyons et al., 2011).

**Table 2:** Table of SNPs and indels between MG1655 and LS5218.

**Location**	**Gene**	**Type**	**CDNA change**	**AA change**
**280113**	insX	CDS	AAGCTG→GGCTA	Lys82fs
**1101543**	csgG	CDS	A→T	Lys48^b^
**1330578**	yciN	CDS	∆G	Ile31fs
**2173360**	gatC	CDS	∆GG	Val306fs
**2210942**	yehQ	CDS	T→G	b615Glu
**2278174**	yejG	CDS	Insert CTGCTGGT	Phe22fs
**2665747**	csiE	CDS	C→T	Gln105^b^
**2867455**	arpoS	CDS	C→T	Gln33^b^
**3130140**	yghO	CDS	A→T	Lys2^b^
**3473612**	arpsG	CDS	T→A	Leu157^b^
**3560455**	glpR	CDS	Insert C	His51fs
**3662700**	amdtF	CDS	C→T	Gln763^b^
**3815879**	arph	CDS	Insert G	Glu224fs
**3951535**	ailvG	CDS	Insert AT	Gln327fs
**290103**	argF	CDS	T→A	Phe68Tyr
**290174**	argF	CDS	TACAGAAGCTTACC→AAGCCAAACTCACT	ValGln40GluAla
**290192**	argF	CDS	ATGGCAAG→GCGGTAAA	Asn36Ser
**290221**	argF	CDS	AC→GA	Gln28Lys
**378700**	afrmA	CDS	T→G	Val291Gly
**579285**	ybcV	CDS	A→G	Ile104Val
**616676**	entF	CDS	C→A	Asp840Glu
**903248**	aartP	CDS	C→A	Leu163Met
**1169836**	ldtC	CDS	T→C	Leu180Pro
**1235101**	fadR	CDS	T→A	Leu55Gln
**1301992**	aoppA	CDS	A→T	Asn271Tyr
**1301999**	aoppA	CDS	G→A	Ser273Asn
**1302190**	aoppA	CDS	A→G	Asn337Asp
**1305442**	aoppD	CDS	T→G	Val230Gly
**1306736**	aoppF	CDS	T→G	Ser325Ala
**1337394**	aacnA	CDS	A→G	Ser522Gly
**1358859**	apuuP	CDS	A→G	Tyr110Cys
**1643679**	aydfU	CDS	T→A	Leu209Gln
**1652331**	aintQ	CDS	T→C	Phe261Leu
**1894839**	apabB	CDS	T→C	Leu12Pro
**2003346**	afliC	CDS	C→A	Asn87Lys
**2040433**	ayedY	CDS	C→A	Ala319Asp
**2322251**	atoC	CDS	T→G	Ile129Ser
**3035546**	prfB	CDS	A→G	Thr246Ala
**3214757**	arpoD	CDS	T→C	Tyr571His
**3300572**	yhbS	CDS	G→A	Asp63Asn
**3388041**	aaaeB	CDS	A→C	Thr50Pro
**3554135**	amalT	CDS	T→A	Trp351Arg
**3725176**	aglyQ	CDS	A→C	Glu48Ala
**4243857**	amalF	CDS	G→T	Gly407Cys
**4300405**	mdtP	CDS	A→T	Gln209Leu
**4342047**	amelA	CDS	T→A	Leu46Gln
**289241**	yagI	Upstream	C→A (−79)	
**289281**	yagI	Upstream	TTGG→CTGA (−119)	
**579146**	nmpC	Upstream	T→C (−2321)	
**579651**	nmpC	Upstream	G→A (−2826)	
**579671**	nmpC	Upstream	A→G (−2846)	
**579717**	nmpC	Upstream	T→G (−2892)	
**579811**	nmpC	Upstream	G→A (−2986)	
**687852**	hscC	Upstream	C→A (−4459)	
**696470**	ybeX	Upstream	G→A (−4686)	
**1299464**	insZ	Upstream	A→C (−4142)	
**1665170**	clcB	Upstream	A→C (−145)	
**1979271**	cheA	Upstream	ATG→TTT (−3947)	
**2118488**	wcaN	Upstream	G→A (−4161)	
**2118495**	wcaN	Upstream	C→A (−4168)	
**2118501**	wcaN	Upstream	TGTGCTCGGGTCTT→AGGTCC (−4175)	
**2118526**	wcaN	Upstream	T→A (−4199)	
**2118560**	wcaN	Upstream	Insert T (−4233)	
**2118599**	wcaN	Upstream	TGTGCTCGGGACC→GCGTACAGATT (−4272)	
**2118649**	wcaN	Upstream	C→T (−4322)	
**2725818**	akgtP	Upstream	T→C (−72)	
**3707947**	dppD	Upstream	G→T (−4099)	
**4035734**	fadB	Upstream	A→C (−4763)	
**4166470**	trmA	Upstream	G→A (−3200)	
**4223638**	arpA	Upstream	A→G (−1151)	
**4296380**	nrfD	Downstream	Insert CG (4948)	
**4510238**	yjhD	Upstream	A→C (−3382)	
**4542681**	nanM	Upstream	∆A (−3917)	

Fs, frameshift.

a Mutation also reported for NCM3722 (Lyons et al., 2011).

b , stop codon.

**Fig. 2:**
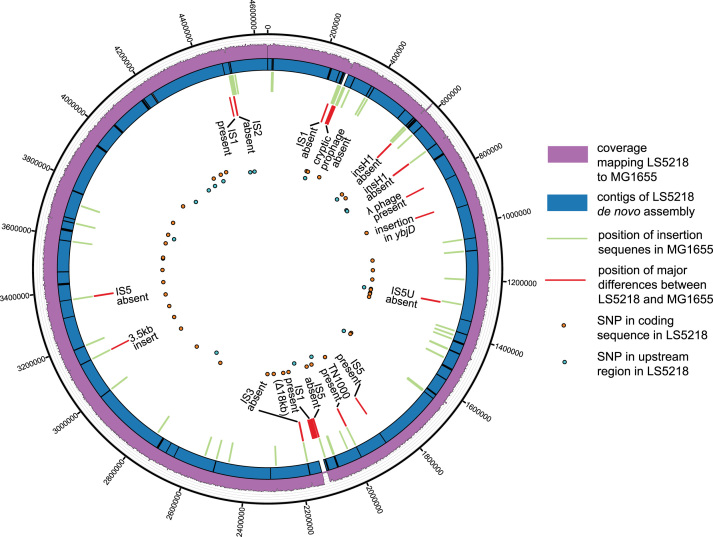
Circular plot of LS5218 features mapped to MG1655. The outer purple histogram displays the coverage of LS5218 sequencing reads as mapped to MG1655. Average coverage was 200× with breaks displayed at genomic regions that differ between the strains. The contigs generated from the LS5218 *de novo* assembly are blue. Many of these breaks correspond to locations of native MG1655 insertion sequences (green bars). The large insertions and deletions of LS5218 are displayed in red and labeled. SNPs are spread throughout with those in coding regions shown in orange and those upstream of genes shown in light blue. (For interpretation of the references to color in this figure legend, the reader is referred to the web version of this article.)

The L55Q mutation in *fadR* replaces a hydrophobic leucine with a hydrophilic glutamine within the DNA binding domain. This change likely affects the interaction of *fadR* with the DNA backbone (van Aalten et al., 2000, Xu et al., 2001). The *atoC* mutation, I129S, is responsible for conferring constitutive expression of the *ato* operon, however the mechanism of this action remains unknown. Beyond the expected mutations, the major insertions and deletions were concentrated around insertion elements whereas the small SNPs were distributed evenly throughout the genome. Coverage of the LS5218 sequence compared to MG1655 (Fig. 2) highlights the position of known insertion elements in MG1655 for comparison of the large and small differences along with the assembled contigs.

**Fig. 3:**
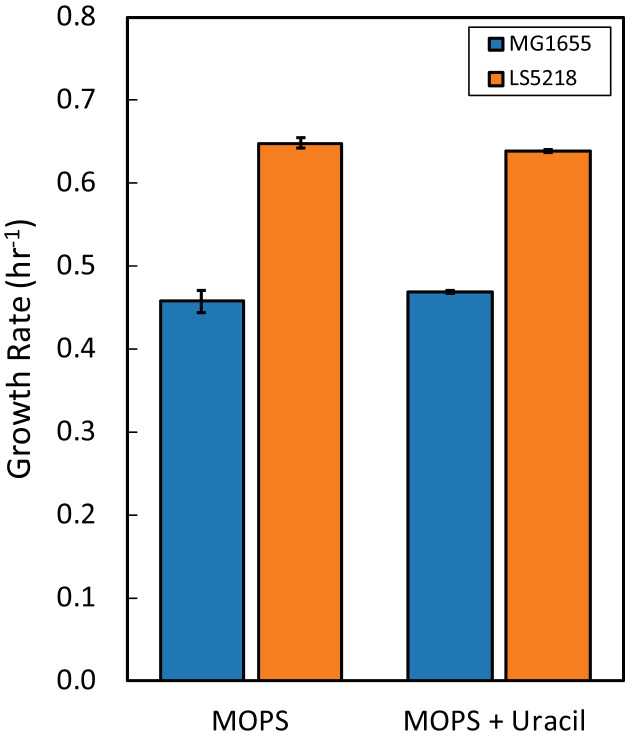
*E. coli* MG1655 and LS5218 growth rate in MOPS minimal media with glucose or glucose supplemented with 20 μg/mL uracil.

We found that LS5218 has numerous insertions, deletions, genomic arrangements, and SNPs as well as the presence of the F plasmid. The highlighted 17 large insertions and deletions as well as the 74 SNPs could affect gene expression beyond the anticipated changes in fatty acid degradation pathways. One of the primary differences between MG1655 and LS5218 is the *rph* mutation. It is known that MG1655 has a frameshift mutation in *rph* that also causes pyrimidine starvation due to polar effects on the downstream *pyrE* gene (Blattner et al., 1997b). MG1655 also has a known frameshift in *ilvG* that affects expression of a branched-chain amino acid biosynthesis operon (Lawther et al., 1982), which is not present in LS5218. The fact that LS5218 does not have these mutations may partially explain why we see increased growth rates in LS5218 when compared with MG1655 on MOPS minimal media (Fig. 3).

During our evaluation of large genomic changes we found a 3.5 kb insert in LS5218 containing three genes putatively annotated for fatty acid and secondary metabolite biosynthesis. These genes have homology towards an acyl-carrier protein (B1R43_RS14595), an aldehyde/flavonoid reductase with an NAD(P) binding site (B1R43_RS14600) and a fatty acyl-AMP ligase (B1R43_14605). None of these enzymes have been studied, but their putative annotations suggest that they could augment fatty acid metabolism with new or enhanced enzymes. Among the small changes compared to MG1655, mutations in *rpoS* (sigma-28) and *rpoD* (sigma-70) could have large pleiotropic effects on the cell. LS5218 also has a mutation in *prfB* (release factor 2), similar to that of *E. coli* BL21. These mutations could explain the differences in gene expression (identified by microarray) between MG1655 and NCM3722, a close LS5218 relative (Soupene et al., 2003). This study showed significantly higher mRNA expression of flagella and chemotaxis and lower expression of galactitol and maltose operon and regulons (Soupene et al., 2003).

## 3 Conclusions

The genome sequence of *E. coli* LS5218 disproves a commonly held assumption about its relationship to the reference K12 strain. LS5218 is a close relative of NCM3722 and not MG1655, with many of the genomic differences reported here also seen in a comparison of the *E. coli* strains MG1655 and NCM3722 (Table 1, Table 2). Unfortunately the strain history for NCM3722 was lost (Lyons et al., 2011, Soupene et al., 2003) so we do not know if they are directly related. We theorize, based off phylogeny and common genetic variations, that NCM3722 and LS5218 share a similar derivation path and are a better representation of the original *E. coli* K-12 isolate than MG1655 (Soupene et al., 2003). The sequence of *E. coli* LS5218 allows us to have a better understanding of the genetic background for this widely used production strain and raises the question whether other mutations, in addition to *fadR601* and *atoC*(*c*), could be contributing to the improved production rates compared to other *E. coli* derivatives (Salamanca-cardona et al., 2014, Tappel et al., 2012, Ushimaru et al., 2015). The additional overlooked differences between LS5218 and MG1655 highlight the fact that genetic background is an important feature when selecting a host for metabolic engineering. The choice may have profound effects on successful engineering and strain performance.

## 4 Materials and methods

DNA was isolated from LS5218 using the Wizard® Genomic DNA Purification Kit (Promega) and sequenced by the University of Wisconsin Biotechnology Center. A paired end library was run on an Illuminia Hi-Seq. 2500. Sequencing generated 5431,968 reads (2 × 250). A *de novo* assembly was created using SPAdes (Bankevich et al., 2012). The draft genome contained 121 contigs (200 bp or greater) with an N50 of 159,470. The genome length was 4699,198 with an average coverage of 279X. The assembly included the complete F plasmid (67,502 bp) and bacteriophage phiX174 (5513 bp). The draft genome was annotated through the NCBI Prokaryotic Genome Annotation Pipeline (PGAP). The genome sequence has been deposited in GenBank under bioproject PRJNA374891 and accession number MVJG00000000. Reads have been deposited to the Sequence Read Archive with accession number SRR5572609.

Sequencing reads (as FASTQ files) of *E. coli* LS5218 were mapped to completed reference genomes *E. coli K12 MG1655* (GCA_000005845.2) and *E. coli* NCM3722 (GCF_001043215.1) using Bowtie2 using the “fast-local” setting (Langmead and Salzberg, 2012). The output sequence alignment map (SAM) file was converted to a binary alignment map (BAM) file and sorted using SAMtools (Li et al., 2009). Variants were then called using FreeBayes (Garrison and Marth, 2012) and Naïve Variant Caller (Galaxy open source bioinformatics tool) (Goto et al., 2011). Variant calls were then annotated using SnpEff (Cingolani et al., 2012b) and variant calls with a quality of less than 30 were sorted out using SnpSift (Cingolani et al., 2012a). Large gaps and insertions were isolated using progressive Mauve alignment with default settings (Darling et al., 2004, Darling et al., 2010) and the pan-genome for the *E. coli* K-12 strains was generated with BPGA (default settings) (Chaudhari et al., 2016).

Specific growth rates calculated from growth curves generated in MOPS minimal media (Neidhardt et al., 1974) supplemented with 0.2 wt% glucose and 20 μg/mL uracil, when indicated. OD600 measurements were taken at 30 min intervals by a Tecan m200.
